# GWAS-by-Subtraction Resolves Coronary Artery Disease-Related and Model-Defined Residual Genetic Components of Ischaemic Stroke

**DOI:** 10.3390/genes17091061

**Published:** 2026-09-01

**Authors:** Binyang Wang, Yuxue Wang, Yong Yin

**Affiliations:** 1 Kunming Medical University, Kunming 650000, China; 20201284@kmmu.edu.cn (B.W.); wangyuxue@kmmu.edu.cn (Y.W.); 2 Yunnan University Affiliated Hospital, Yunnan University, Kunming 650000, China

**Keywords:** ischaemic stroke, coronary artery disease, GWAS-by-subtraction, genomic structural equation modelling, genetic pleiotropy

## Abstract

**Background/Objectives:** Genetic overlap between any ischaemic stroke (AIS) and coronary artery disease (CAD) can obscure associations less closely tied to coronary risk. We aimed to separate coronary-shared from residual AIS genetic effects. **Methods:** We applied GWAS-by-subtraction to GIGASTROKE AIS and UK Biobank/CARDIoGRAMplusC4D CAD summary statistics. A Cholesky model separated a CAD-related shared component (F1) from a model-defined residual AIS component (F2). We tested model order, factor covariance, population prevalence, CAD input, LD reference and sample-size parameter then characterised loci using FUMA, SuSiE-RSS, genetic correlation, MAGMA, SMR/HEIDI and targeted colocalisation. **Results:** AIS and CAD had a genetic correlation of 0.490. F1 and F2 had SNP heritabilities of 0.0251 and 0.00414, and F2 contained 14 FUMA loci. F2 Z scores remained correlated across covariance (minimum r = 0.994), prevalence (minimum r = 0.998) and alternative-CAD analyses (r = 0.984). Reverse ordering produced reallocation of source-trait signal. At rs974819, the T allele was associated positively with CAD and negatively with marginal AIS, producing a model-derived CAD-AIS-discordant F2 association. Source-trait colocalisation showed that NBEAL1, PHACTR1 and PDGFD signals were present in one or both input-trait analyses, whereas the F11 protein signal aligned with AIS and F2. In a potentially overlapping FinnGen cross-implementation analysis, all 13 F2 lead variants showed concordant directions. Within overlapping stroke-subtype analyses, F2 showed stronger alignment with cardioembolic stroke. **Conclusions:** GWAS-by-subtraction resolved a model-defined residual AIS component that was stable across covariance, prevalence and CAD-input specifications, supporting a component-based view of AIS genetic effects after modelling shared coronary architecture.

## 1. Introduction

Stroke caused 7.3 million deaths and 160.5 million disability-adjusted life-years worldwide in 2021, with ischaemic stroke accounting for most incident events [1]. Ischaemic stroke encompasses several vascular processes. Large-artery atherosclerosis, cardiac embolism and cerebral small-vessel disease all lead to infarction yet differ in origin, proximal mechanism and risk-factor profile. Large genome-wide association studies (GWAS) have expanded the catalogue of stroke loci and shown that subtype-aware analyses recover signals diluted by broad stroke definitions [2,3]. Studies of lacunar stroke have likewise identified contributions from cerebral small-vessel biology beyond conventional atherosclerotic risk [4]. Any ischaemic stroke (AIS) offers greater statistical power, but its summary association profile combines partially overlapping mechanisms.

Coronary artery disease (CAD) contributes substantially to this overlap. CAD and AIS share atherosclerotic, haemodynamic and metabolic determinants, and both are represented in large population cohorts. CAD GWAS have identified hundreds of associations involving lipid metabolism, blood pressure, inflammation, thrombosis and arterial-wall biology [5,6]. Lipid effects also vary across stroke subtypes: LDL cholesterol is more strongly related to large-artery stroke than to cardioembolic or small-vessel stroke, whereas blood-pressure effects are distributed more broadly [7,8]. A conventional AIS GWAS therefore mixes variants acting through CAD-related cardiometabolic architecture with effects less closely tied to coronary disease. Although valuable for locus discovery, the aggregate profile cannot show which associations track shared coronary risk and which remain after that architecture is modelled.

Genomic structural equation modelling (Genomic SEM) provides a summary-statistic framework for estimating multivariate genetic covariance and variant effects on latent dimensions [9]. GWAS-by-subtraction uses this framework to partition a target phenotype into a component shared with a genetically correlated trait and a residual component defined by the fitted model [10]. Unlike simple covariate adjustment or post hoc removal of genome-wide significant CAD loci, the method operates across the polygenic genome and incorporates the sampling covariance of the contributing GWAS. Applied to AIS and CAD, it can estimate variant effects on a CAD-related shared component and on the remaining AIS component while retaining uncertainty due to participant overlap and correlated estimation error.

We applied GWAS-by-subtraction to separate AIS genetic architecture into a CAD-related shared component (F1) and a model-defined residual AIS component (F2). Robustness analyses varied the factor covariance and replaced the primary CAD GWAS. We defined loci with frozen online FUMA analyses, fine-mapped the F2 regions using signed reference-panel LD and compared component correlations with nine vascular and stroke traits through covariance-aware contrasts. A secondary analysis examined atrial fibrillation. Gene, tissue and expression-informed analyses were complemented by targeted colocalisation with arterial or cortical expression and circulating coagulation proteins. We then reimplemented the subtraction structure in FinnGen R13 and quantified its cohort-level overlap with the primary GIGASTROKE analysis. Throughout this manuscript, F2 denotes residual genetic variation conditional on the fitted Cholesky model.

## 2. Materials and Methods

### 2.1. Study Design

**Figure 1:**
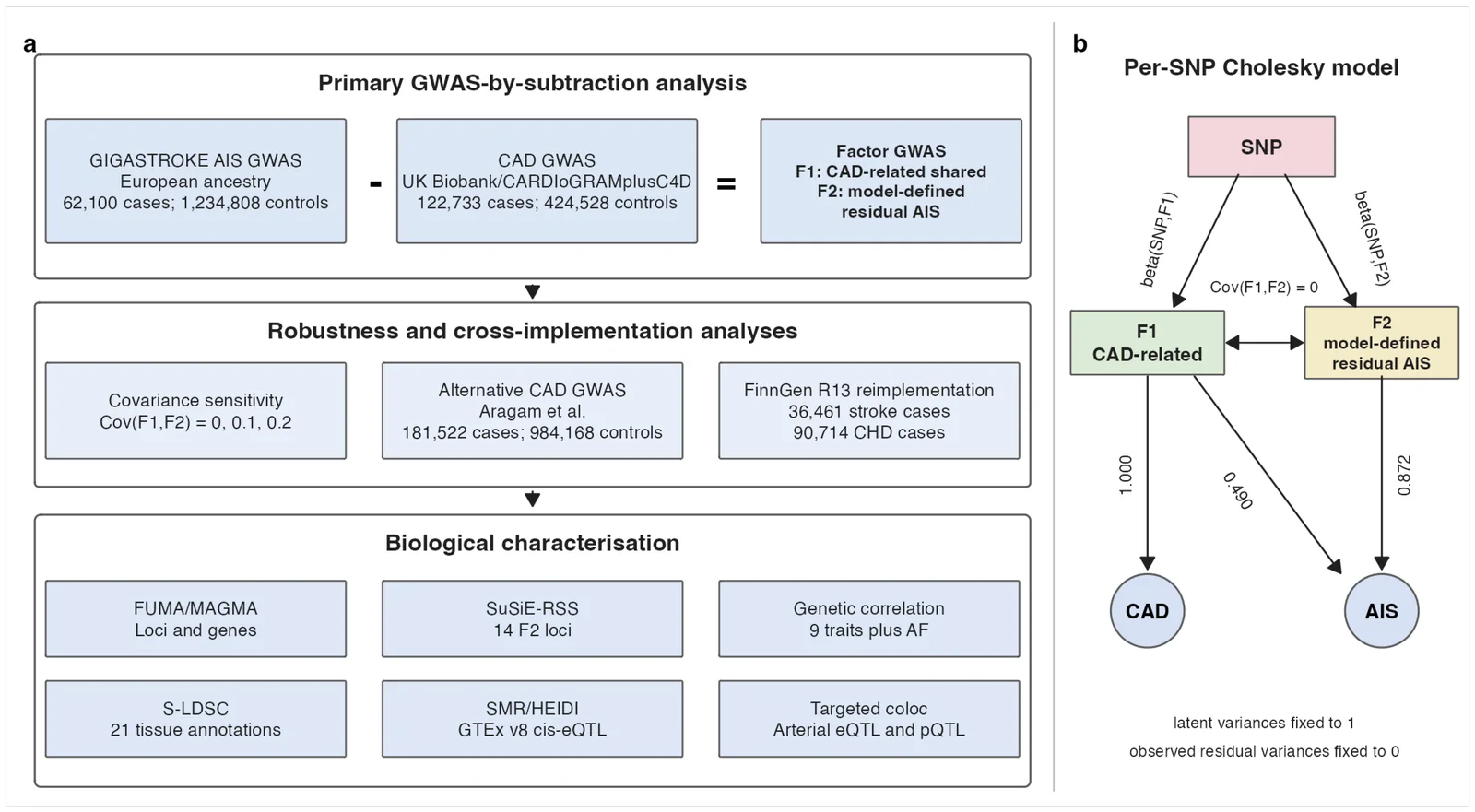
Study workflow and subtraction model. (**a**) Primary GWAS-by-subtraction analysis, robustness analyses and biological characterisation. The minus and equals signs summarise model-based partitioning rather than arithmetic subtraction of association estimates [6]. (**b**) Per-SNP Cholesky model. SNP effects were estimated on F1 and F2; arrows from the factors to observed traits show standardised loadings. The primary model fixed Cov(F1,F2) to zero, latent variances to one and observed residual variances to zero. F1 denotes the CAD-related shared component; F2 denotes the model-defined residual AIS component.

We used GWAS-by-subtraction to separate two genetic components of any ischaemic stroke (AIS). The first, a CAD-related shared component (F1), captured genetic effects shared with coronary artery disease (CAD). The second, a model-defined residual AIS component (F2), captured AIS genetic variation remaining after fitting F1. We characterised both components through locus annotation, genetic correlation, tissue enrichment and expression-informed association analyses and statistically fine-mapped the F2 loci. The same subtraction structure was reimplemented using FinnGen R13 summary statistics for a cross-implementation comparison (Figure 1).

All analyses used publicly available, de-identified GWAS summary statistics. No individual-level participant data were accessed, and no new participants were recruited. Ethical approval and informed consent were obtained by the contributing studies under their original protocols.

### 2.2. Primary GWAS Summary Statistics (Table 1)

The AIS input was the European-ancestry analysis from GIGASTROKE, accession GCST90104540 [3]. This analysis included 62,100 cases and 1,234,808 controls, giving a total sample size of 1,296,908. The downloaded harmonised file used GRCh37 coordinates but did not contain rsIDs. We assigned rsIDs by unique chromosome-position matches to the HapMap3 coordinate reference. The identity of GCST90104540 as AIS was verified against the GWAS Catalog application programming interface.

The CAD input was the European-ancestry meta-analysis by van der Harst and Verweij, accession GCST005194 [5]. It included 122,733 cases and 424,528 controls, for a total sample size of 547,261. The study combined UK Biobank and CARDIoGRAMplusC4D data and used GRCh37 coordinates. The source file did not provide per-variant sample sizes, so we used the reported total sample size throughout.

**Table 1:** GWAS datasets used in the analyses.

Phenotype	Source	ID	Ancestry	Cases	Controls	Total N
Any ischaemic stroke	GIGASTROKE [3]	GCST90104540	European	62,100	1,234,808	1,296,908
Coronary artery disease	van der Harst and Verweij 2018 [5]	GCST005194	European	122,733	424,528	547,261
Stroke excluding subarachnoid haemorrhage	FinnGen R13 [11]	I9_STR	Finnish	36,461	446,552	483,013
Coronary heart disease	FinnGen R13 [11]	I9_CHD	Finnish	90,714	409,472	500,186

### 2.3. Reference Data and Summary-Statistic Harmonisation

We used European LD scores from 1000 Genomes Project phase 3 for LDSC and Genomic SEM [12]. The LD-score reference contained 1,290,028 variants across autosomes. The HapMap3 reference list contained 1,275,314 SNPs [13]. Its alleles were obtained from the corresponding 1000 Genomes European quality-controlled reference genotypes.

Summary statistics were harmonised in Genomic SEM. We restricted analyses to autosomal HapMap3 variants and applied a minor allele frequency threshold of 0.01. An INFO threshold of 0.6 was applied when an INFO field was available. The CAD and derived factor files lacked INFO fields, so INFO filtering was not applied to those datasets.

Harmonisation retained 1,266,791 AIS variants and 1,272,175 CAD variants. Three CAD variants with incompatible alleles were removed during reference alignment, leaving 1,254,765 variants shared by both traits and the reference panel. Binary-trait effects were supplied on the log-odds scale with their standard errors.

### 2.4. Multivariable LD Score Regression

We estimated SNP heritabilities, genetic covariance and genetic correlation using multivariable LD score regression in Genomic SEM [14]. The model used the European LD-score files as both the reference and regression-weight inputs. Sampling prevalences were 0.04788 for AIS and 0.22427 for CAD. Population prevalences were set to 0.10 and 0.07, respectively, for liability-scale covariance estimation.

Sampling prevalences remained fixed at the observed case fractions in every sensitivity run. Only population prevalence was varied in a 3 × 3 grid: 0.05, 0.10 and 0.15 for AIS, and 0.03, 0.07 and 0.12 for CAD. This prespecified range surrounded the primary assumptions and was not intended to reproduce age- or sex-specific lifetime-risk estimates, which are reported on different epidemiological scales [15,16]. Each grid point was evaluated for finite estimates, covariance-matrix status, complete SNP output, genome-wide F2 Z-score correlation and significant-set overlap with the primary setting.

The LDSC sampling covariance matrix retained uncertainty arising from shared participants and other correlated estimation errors. This was important because both primary GWAS datasets included UK Biobank contributions. The cross-trait LDSC intercept was 0.0378 (s.e. 0.0058), consistent with non-zero sampling covariance.

### 2.5. GWAS-by-Subtraction Model

We fitted a two-factor Cholesky decomposition using Genomic SEM v0.0.5 [9]. F1 loaded on CAD and AIS, whereas F2 loaded only on AIS. Both latent variances were fixed to one, and the residual variances of CAD and AIS were fixed to zero. The F1–F2 covariance was fixed to zero in the primary model.

The model can be written as:

CAD = λ(CAD, F1) × F1, AIS = λ(AIS, F1) × F1 + λ(AIS, F2) × F2, Cov(F1, F2) = 0.(1)

Genomic SEM estimated each SNP’s effects on both latent factors. We extracted the factor-specific coefficient, standard error, Wald statistic and two-sided *p* value. The two-trait decomposition was just identified, so global fit indices and an informative Q-SNP heterogeneity test were not available. Model robustness was instead evaluated by relaxing the F1–F2 covariance constraint.

Historical Genomic SEM output files contained a verified column-label offset in the raw-trait fields. Factor estimates, standard errors, Wald statistics, *p* values and reference frequencies were unaffected. We rebuilt canonical files by joining rsIDs to the GRCh37 coordinate reference and restoring the factor effect and non-effect alleles. Each canonical factor file contained 1,254,765 unique variants with valid alleles and coordinates.

### 2.6. Factor Quality Control and Covariance Sensitivity Analysis

We re-harmonised the F1 and F2 summary statistics to HapMap3 and estimated their observed-scale SNP heritabilities and LDSC intercepts. A constant sample-size field of 1,296,908 was used for this LDSC formatting step because latent factors have no natural per-variant sample size. It was not interpreted as an effective factor sample size.

We refitted the genome-wide model with F1–F2 covariance values of −0.2, −0.1, 0, 0.1 and 0.2. For the new negative-covariance settings, an analytic estimator of the same saturated Cholesky model was first validated against the archived iterative Genomic SEM run. We also reversed the trait order, fitting AIS first to obtain an AIS-related shared construct (R1) and a model-defined residual CAD construct (R2). Robustness was quantified by genome-wide Pearson correlations of SNP Z scores, counts of *p* < 5 × 10^−8^ variants and Jaccard similarity of significant sets.

### 2.7. Effective Sample-Size Reporting

For each binary source GWAS, we calculated the case-control effective sample size as Neff = 4/(1/Ncase + 1/Ncontrol). This gave Neff values of 236,506 for GIGASTROKE AIS, 380,832 for the primary CAD GWAS and 613,021 for the Aragam CAD sensitivity GWAS (Table S5). Because a latent-factor GWAS has no natural case-control count, we used 236,506 as a conservative power reference for F2 rather than assigning a formally derived factor Neff. The constant N of 1,296,908 in LDSC-formatted factor files served only as a summary-statistic formatting field.

### 2.8. Alternative CAD GWAS Sensitivity Analysis

We repeated multivariable LDSC, harmonisation and the primary orthogonal subtraction model after replacing the van der Harst and Verweij CAD input with the predominantly European Aragam et al. 2022 CAD GWAS [5,6]. The alternative CAD dataset included 181,522 cases and 984,168 controls. Its sampling prevalence was calculated from these counts, and the CAD population prevalence remained 0.07. Harmonisation produced 1,257,393 shared SNPs for Genomic SEM.

The sensitivity comparison evaluated the number of genome-wide significant F2 variants and six sentinel SNPs present in the archived primary-analysis sentinel file. These variants were rs2351524, rs2723334, rs6536024, rs10774625, rs974819 and rs11072793. For each SNP, we compared effect direction and two-sided Wald *p* values between the primary and alternative-CAD F2 analyses. This test assessed dependence on the selected CAD GWAS; it did not redefine the 14 FUMA loci from the frozen primary F2 job.

### 2.9. Locus Definition and Functional Annotation

Canonical F1 and F2 summary statistics were analysed through the online FUMA SNP2GENE platform v1.8.2 [17]. The frozen jobs were 759,333 for F1 and 759,225 for F2. Both jobs used GRCh37, 1000 Genomes phase 3 European LD and a genome-wide threshold of *p* < 5 × 10^−8^.

Independent significant SNPs were defined at r2 < 0.6. Lead SNPs were defined at r2 < 0.1, and risk loci separated by less than 250 kb were merged. Candidate SNPs were taken from the European reference panel when they had *p* < 0.05 and were linked to an independent significant SNP. The extended MHC exclusion setting was enabled.

Positional mapping assigned protein-coding genes within 10 kb using Ensembl v102. FUMA eQTL mapping and chromatin-interaction mapping were disabled. Therefore, all reported FUMA mapped genes arose from positional mapping.

MAGMA v1.08 was run within FUMA [18]. Gene analysis tested 17,512 protein-coding genes without an added gene window. Competitive gene-set analysis tested 17,001 sets. Tissue-expression analysis tested 54 GTEx v8 tissues while conditioning on average expression. Bonferroni thresholds were calculated within each reported MAGMA family.

To evaluate the artificial constant-N field, we repeated a paired local MAGMA v1.08 analysis with the archived SNP set, locally available NCBI37.3 gene annotation and boundaries, 1000 Genomes European LD reference, SNP-wise mean model and multiple-testing rule held fixed. Only the SNP-level N value changed within this paired local workflow, from 1,296,908 to the conservative AIS case-control effective sample size of 236,506. The latter was treated as a sensitivity parameter, not an estimate of F2 effective sample size. The frozen online FUMA analysis used its Ensembl-based annotation and was not redefined by this local test.

### 2.10. Fine-Mapping Analysis

We fine-mapped all 14 FUMA-defined F2 loci with SuSiE-RSS v0.14.2. For each locus, we extracted corrected F2 effect estimates and standard errors within 500 kb of the lead SNP and calculated effect-allele-aligned Z scores. Signed LD matrices were calculated from effect-allele dosages in 503 European participants from the 1000 Genomes Project phase 3 GRCh37 reference panel [12]. We retained biallelic variants with an exact allele match to F2, reference-panel minor allele frequency ≥ 0.01 and non-zero genotype variance.

SuSiE-RSS was fitted with a maximum of ten single effects and 95% credible-set coverage. Because F2 is a latent component without a formally defined factor effective sample size, the primary analysis used Z scores and signed LD without supplying the constant summary-statistic N field. A ridge of 10^−4^ was applied to each singular reference LD matrix. Credible sets were retained when their minimum absolute within-set correlation met the SuSiE purity threshold of 0.5. Posterior inclusion probabilities (PIPs) ≥ 0.10 were reported descriptively; this threshold did not define credible-set membership. We recorded convergence, iteration count and the LD-mismatch diagnostic for every locus.

LD-reference sensitivity was evaluated at NBEAL1, FGA/FGG, F11/KLKB1, PHACTR1 and the rs974819/PDGFD region using the public PolyFun UKBB_LD resource [19]. The resource contains signed, unphased genotype-correlation matrices from approximately 337,000 British-ancestry UK Biobank participants in 2763 overlapping 3 Mb GRCh37 blocks with 1 Mb start increments. Files were downloaded on 28 August 2026 from https://data.broadinstitute.org/alkesgroup/UKBB_LD; the resource has no versioned release identifier, so the five selected blocks are identified by URL and SHA-256 checksum in Table S14. The preparation code was archived at PolyFun commit 8b17b4ec87fde11f8d216c049c08d1420cd25de6. No additional sparsification threshold was applied; absent entries in the public sparse matrices were treated as zero. Alleles, coordinates and SNP order were aligned to F2. Sparse reconstruction produced minimum eigenvalues of −0.0015 to −0.0045, so we applied the prespecified ridge transformation R* = (1 − 10^−4^)R + 10^−4^I. The matrices remained slightly non-positive-semidefinite; susie_rss in susieR 0.14.2 then reported setting negative eigenvalues to zero internally. Runs used L = 10, 95% coverage, fixed residual variance, max_iter = 1000 and tol = 10^−3^. We compared signed-LD correlations, top SNPs and PIPs, credible-set number and size, credible-set overlap and purity. Both references were restricted by the HapMap3 variants available in the factor GWAS.

### 2.11. Assessment of Locus Novelty

We classified the 14 F2 lead loci against prior stroke and cerebrovascular associations. A locus was considered known when the lead or a European r2 ≥ 0.8 proxy had a genome-wide significant prior association. Evidence came from the FUMA GWAS Catalog snapshot dated 29 November 2022 and primary stroke GWAS reports.

For rs974819, the only locus without prior stroke evidence in the archived snapshot, we performed two additional checks. First, we queried the current GWAS Catalog API on 2 August 2026 for rs974819 and six validated European r2 ≥ 0.8 proxies. Second, we scanned a +/−500 kb window around the lead in eight prespecified stroke or cerebrovascular summary-statistic datasets.

The regional datasets comprised MEGASTROKE, GIGASTROKE AIS, three GIGASTROKE AIS subtypes, FinnGen stroke, FinnGen embolic stroke and FinnGen transient ischaemic attack. The Aragam CAD analysis was used as a positive-control regional scan. We recorded absence of prior evidence only when complete searches returned zero stroke or cerebrovascular associations at *p* < 5 × 10^−8^. This procedure assessed reporting novelty at the genome-wide threshold; conditional association structure was outside its scope.

### 2.12. FinnGen Construct Reimplementation and Cohort-Overlap Assessment

We implemented the same subtraction model using FinnGen R13 I9_STR and I9_CHD summary statistics [11]. FinnGen coordinates were lifted from GRCh38 to GRCh37 with the UCSC chain file. We retained unique autosomal rsIDs and defined the alternate allele as the effect allele. Harmonisation retained 1,246,100 stroke variants and 1,246,103 CHD variants. The FinnGen Genomic SEM input contained 1,259,947 shared variants.

The FinnGen LDSC covariance matrix was fitted with the same population prevalence settings used for the primary analysis. Factor starting values were derived from the FinnGen genetic covariance matrix. The primary FinnGen model fixed the F1–F2 covariance to zero and generated a FinnGen F2 construct.

Cross-implementation testing started with exact matching of each FUMA F2 lead SNP. If a lead was unavailable, we searched FUMA candidate variants explicitly linked to that lead at European r2 ≥ 0.8. Alleles and effect-allele frequencies were aligned before comparing effects.

We reported direction consistency and two graded association criteria: same direction with *p* < 0.05 and same direction with *p* < 0.001. A two-sided exact binomial test evaluated whether the direction-consistency rate exceeded the 0.5 null expectation. The target of comparison was the F2 subtraction construct fitted in each dataset.

We assessed cohort-level overlap against the official GIGASTROKE cohort table [3]. FinnGen contributed 8851 AIS cases and 174,426 controls to the European GIGASTROKE meta-analysis. These counts represented 14.3% of GIGASTROKE cases and 14.1% of GIGASTROKE controls and, relative to the R13 endpoint counts, potential overlap of up to 24.3% of cases and 39.1% of controls. Exact participant-level overlap across releases and phenotype definitions was unavailable; the FinnGen analysis was therefore designated a cross-implementation comparison.

### 2.13. Genetic Correlations and Formal Component Contrasts

We prespecified nine comparison traits, comprising six external vascular traits and three overlapping GIGASTROKE stroke-subtype analyses: systolic blood pressure, diastolic blood pressure [20], LDL cholesterol, HDL cholesterol, triglycerides [21], white matter hyperintensities [22], LAS, CES and SVS [3]. GWAS accessions and sample sizes are provided in Table S2.

For each external trait, we jointly analysed F1, F2 and the external trait through three-trait multivariable LDSC. This produced one genetic covariance matrix and one complete sampling covariance matrix per trait. Factor alleles were validated before analysis.

We obtained each genetic correlation as the relevant covariance divided by the square root of both SNP heritabilities. The formal contrast was Delta = rg(F2) − rg(F1). Its standard error was calculated by the delta method using the complete Genomic SEM sampling covariance matrix in the verified three-trait order.

Contrast *p* values were adjusted across the nine prespecified traits using the Benjamini–Hochberg procedure. Results with external-trait heritability Z < 4 or genetic correlations outside the parameter boundary were labelled exploratory.

LAS, CES and SVS were constituent GIGASTROKE subtype analyses and shared participants with the AIS input. Their genetic correlations were therefore interpreted as descriptive part-whole comparisons of subtype alignment within the stroke dataset, not as independent external validation.

In a mechanism-oriented secondary analysis, we jointly analysed F1, F2 and atrial fibrillation (AF) using the same three-trait LDSC and delta-method procedure. The AF GWAS included 65,446 cases among more than 500,000 participants [23]. We tested one additional contrast, Delta = rg(F2,AF) − rg(F1,AF), and report its unadjusted two-sided *p* value in Table S6.

### 2.14. Stratified LD Score Regression

We used S-LDSC v1.0.1 to evaluate a targeted panel of 21 GTEx tissue annotations [24,25]. The panel contained 3 arteries, 2 cardiac tissues, liver, skeletal muscle, whole blood and 13 brain tissues. Tissue LD scores were computed chromosome-wise from quality-controlled GRCh37 genotypes of 489 European participants in 1000 Genomes Project phase 3, using a 1 cM LD window and HapMap3 SNPs outside the MHC.

Factor summary statistics were analysed in the cell-type-specific heritability framework with the baselineLD model, HapMap3 regression weights and the no-MHC SNP set. Overlapping annotations were enabled, and tissue coefficients were estimated. Benjamini–Hochberg correction was applied separately to the 21 tissue coefficients for F1 and F2; direct cross-factor coefficient contrasts were outside the prespecified analysis.

### 2.15. SMR and HEIDI Analysis

We integrated the factor GWAS with GTEx v8 cis-eQTL summary data [25] using SMR v1.3.1 [26]. Analyses included aorta, coronary artery, tibial artery and seven selected brain tissues. The brain panel comprised amygdala, anterior cingulate cortex, cerebellum, cortex, frontal cortex BA9, hippocampus and substantia nigra.

The SMR reference consisted of 503 European participants from the 1000 Genomes quality-controlled panel. Factor GWAS files contained 1,254,765 variants. We used the default SMR instrument-selection and HEIDI settings.

Across both factors and all 10 tissues, 61,354 tissue-gene tests were evaluated. Strict results required *p*(SMR) < 0.05/61,354 = 8.149 × 10^−7^ and *p*(HEIDI) > 0.01. These criteria identify expression–trait associations compatible with a shared local variant and without detected HEIDI heterogeneity.

### 2.16. Targeted Colocalisation Analysis

GTEx v8 cis-eQTL data [25] were examined in aorta, coronary artery, tibial artery and brain cortex. Protein analyses used F11 levels from GCST90469165 (N = 47,745) [27] and fibrinogen from GCST90019421 (N = 10,708) [28]. We then used approximate Bayes-factor colocalisation [29] to evaluate local association patterns for nine genes at selected F2 loci: NBEAL1, PHACTR1, F11, KLKB1, FGA, FGB, FGG, PDGFD and ADAMTS7. Each analysis used F2 variants within 500 kb of the locus lead. Variants were matched by rsID and allele pair; QTL effects were reversed when the effect alleles were opposite, and high-frequency palindromic variants were removed.

For the original F2-QTL analyses, both F2 and the molecular trait were specified as quantitative traits. In the source-trait analyses, AIS and CAD were specified as case-control traits using their respective case fractions, whereas F1 and F2 were specified as standardised quantitative constructs. Approximate Bayes-factor colocalisation used prior probabilities p1 = 10^−4^, p2 = 10^−4^ and p12 = 5 × 10^−6^. We report posterior probabilities for distinct local signals (PP3) and a shared local signal (PP4). Because the model assumes at most one causal variant per trait in a region, PP4 was interpreted as compatibility with a shared local association signal rather than proof of one causal variant. Prior sensitivity was evaluated by identifying the smallest p12 for which PP4 exceeded 0.75. Full results are provided in Table S9.

For each of the 38 archived F2-QTL combinations, we repeated colocalisation with the primary AIS and CAD source GWAS and with F1. Each four-way comparison used the same +/−500 kb region, allele-matched common SNP set and priors. Because the four GWAS are statistically dependent, PP4 patterns were interpreted descriptively rather than as independent contrasts or evidence of F2-specificity.

### 2.17. Statistical Analysis and Software

Unless stated otherwise, all association tests were two-sided. Genome-wide significance was *p* < 5 × 10^−8^. MAGMA gene, tissue and gene-set analyses used the one-sided competitive or regression tests supplied by FUMA. We used family-specific Bonferroni correction for MAGMA and SMR, and Benjamini–Hochberg correction for prespecified correlation contrasts and S-LDSC. Colocalisation was targeted and evaluated using posterior probabilities and prior-sensitivity analysis rather than frequentist multiplicity correction.

Analyses were performed in R 4.5.2 (Genomic SEM 0.0.5, lavaan 0.7-2, data.Table 1.18.2.1, susieR 0.14.2, coloc 5.2.3 and ggplot2 4.0.2). Gene-based tests and functional annotation used FUMA SNP2GENE v1.8.2 with MAGMA v1.08.

## 3. Results

### 3.1. GWAS-by-Subtraction Separated Two Heritable AIS Components

AIS and CAD showed a genetic correlation of 0.4898 (s.e. 0.0392), supporting a decomposition into shared and residual components. The standardised loadings were 0.490 from F1 to AIS, 1.000 from F1 to CAD and 0.872 from F2 to AIS. These loadings defined F1 as the CAD-related shared component and F2 as the model-defined residual AIS component (Figure 1).

All 1,254,765 SNP models converged for both factors. F1 had SNP heritability 0.02509 (s.e. 0.00175; Z = 14.33), whereas F2 had SNP heritability 0.004144 (s.e. 0.000418; Z = 9.91). The LDSC intercepts were 0.9916 for F1 and 1.0047 for F2. Downstream bivariate LDSC estimated rg(F1,F2) = −0.0744 (s.e. 0.0464; *p* = 0.109), consistent with the model constraint.

**Figure 2:**
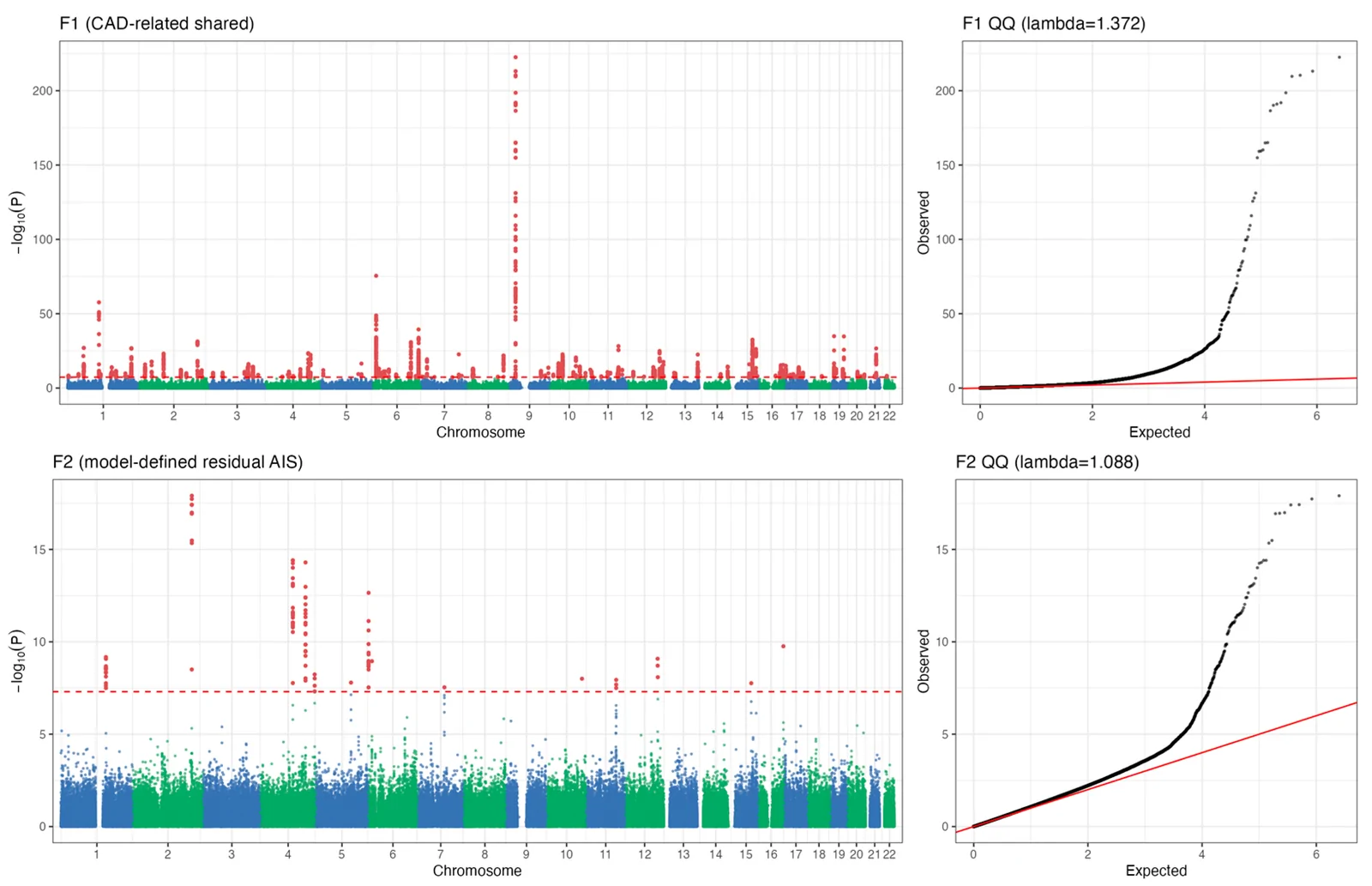
Genome-wide association results for F1 and F2. Manhattan and quantile–quantile plots show two-sided Wald *p* values for 1,254,765 variants. The dashed line marks *p* = 5 × 10^−8^. Red points denote genome-wide significant variants. The LDSC intercepts were 0.9916 for F1 and 1.0047 for F2.

F1 contained 2278 genome-wide significant variants, compared with 96 for F2 (Figure 2 and Table 2). Across fixed F1–F2 covariance values from −0.2 to 0.2, F2 produced 96 to 111 significant variants. Genome-wide F2 Z-score correlations with the orthogonal primary model ranged from 0.9943 to 0.9987, and significant-set Jaccard similarity ranged from 0.725 to 0.913 (Table S1). F1 remained effectively unchanged, with Z-score correlations above 0.99999998.

Reverse ordering yielded 156 significant SNPs for the AIS-first shared construct R1 and 579 for the residual-CAD construct R2. Forward F2 correlated strongly with R1 (r = 0.956) and inversely with R2 (r = −0.780), quantifying the expected order-dependent reallocation of shared and residual signal (Table S10). Across the nine population-prevalence settings, all 1,254,765 SNP models were complete, both covariance matrices passed the archived status checks, F2 Z-score correlations with the primary setting ranged from 0.9982 to 1.0000 and 95 to 101 variants were genome-wide significant (Table S11).

### 3.2. An Alternative CAD GWAS Preserved the Sentinel F2 Signals

Substituting the Aragam et al. CAD GWAS gave AIS-CAD rg = 0.5072 (s.e. = 0.0425) and produced 112 genome-wide significant F2 variants, compared with 96 in the primary analysis. All six prespecified sentinel SNPs retained the same effect direction and remained genome-wide significant. Across these variants, sensitivity-analysis *p* values ranged from 3.83 × 10^−22^ for rs2351524 to 2.36 × 10^−8^ for rs10774625 (Table S7). The previously unreported rs974819 F2 association strengthened from *p* = 1.16 × 10^−8^ to *p* = 5.63 × 10^−10^. The sentinel signals were therefore robust to the choice of CAD input. This analysis left the 14 frozen FUMA loci unchanged.

### 3.3. FUMA Identified 14 Risk Loci for F2

FUMA identified 503 independent significant SNPs, 215 lead SNPs and 138 risk loci for F1. These loci contained 20,980 candidate SNPs and 449 positionally mapped genes. F2 contained 24 independent significant SNPs, 14 lead SNPs and 14 risk loci. The corresponding loci contained 1085 candidate SNPs and 42 positionally mapped genes (Table 2).

The strongest F2 association was rs35212307 at 2q33.1 (beta = −0.331; s.e. 0.0376; *p* = 1.24 × 10^−18^). Other prominent signals mapped near the fibrinogen cluster, FOXF2, PHACTR1, GRK5 and SH2B3. The complete 14-locus set is shown in Table 3.

Twelve F2 loci had a genome-wide significant prior stroke association in high LD. The PHACTR1 locus led by rs9349379 was classified as a known cerebrovascular locus. rs974819 was the only previously unreported genome-wide significant F2 association, but it arose within an established CAD locus. Effect estimates are aligned to A1. Positional genes were assigned within 10 kb.

### 3.4. Fine-Mapping Resolved Several F2 Loci but Not Rs974819

All 14 signed-LD SuSiE-RSS models converged within two to ten iterations. The regional analyses included 145 to 528 allele-aligned variants. Thirteen loci produced one purity-qualified 95% credible set; rs974819 produced no set meeting the purity criterion. Four loci had single-variant credible sets: rs12524544 near FOXF2, rs9349379 at PHACTR1, rs10886430 near GRK5 and rs12445022 at 16q24.2. Their top PIPs ranged from 0.978 to 0.999 (Table S8).

Resolution differed across the coagulation-related regions (Table 4). The FGA/FGG locus had a two-variant credible set containing rs6825454 and rs1025154; rs6825454 had PIP = 0.913. The F11/KLKB1 locus retained a five-variant credible set led by rs2289252 (PIP = 0.594), whereas the FUMA lead rs1511802 had PIP = 0.150. At 11q22.3, rs974819 had the highest PIP (0.390), followed by rs2839812 (0.227) and rs2128739 (0.153), but the regional signal did not yield a purity-qualified credible set. Fine-mapping therefore narrowed several loci while leaving the previously unreported F2 association unresolved. The complete 14-locus summary is provided in Table S8.

At the five key loci, signed-LD correlations between the 1000 Genomes and UK Biobank references ranged from 0.963 to 0.994. The same top SNP was retained at NBEAL1, FGA/FGG, PHACTR1 and rs974819/PDGFD. At F11/KLKB1, the top PIP shifted from rs2289252 (0.594) to rs1511802 (0.396), and the UK Biobank analysis produced no purity-qualified credible set. PHACTR1 remained a single-variant credible set, and rs974819 remained unresolved (Table S14).

### 3.5. rs974819 Marked a CAD-Discordant F2 Association at 11q22.3

The European r2 ≥ 0.8 proxy set for rs974819 contained seven variants. The archived FUMA Catalog snapshot returned 26 records at this locus, none for stroke or cerebrovascular phenotypes. The current GWAS Catalog API returned 52 proxy-association combinations, including 50 genome-wide significant associations. None involved stroke or a cerebrovascular phenotype through 2 August 2026.

No variant within +/−500 kb reached *p* < 5 × 10^−8^ in any of the eight prespecified stroke-related datasets. The same region contained 353 significant variants in the Aragam CAD positive control, with a minimum *p* value of 1.90 × 10^−33^. These searches classified rs974819 as a previously unreported F2 association within an established CAD region.

All effects were aligned to the T allele (Table 5 and Table S12). The primary CAD estimate was positive (beta = 0.0614; s.e. = 0.0055; *p* = 1.12 × 10^−28^), whereas the marginal GIGASTROKE AIS estimate was negative (beta = −0.0282; s.e. = 0.0081; *p* = 5.17 × 10^−4^). Primary F1 and F2 coefficients were 0.121 and −0.162, respectively. The Aragam-CAD and alternative-F2 estimates showed the same positive-negative contrast. FinnGen stroke, coronary heart disease and F2 estimates were −0.0414, 0.0505 and −0.195, respectively. Raw-disease betas are log-odds effects, whereas factor betas are standardised latent coefficients and are compared by direction rather than absolute magnitude.

An indicative fixed-effect combination of the GIGASTROKE and FinnGen stroke estimates gave beta = −0.0335 (s.e. 0.00627; *p* = 8.99 × 10^−8^). Cochran’s Q was 1.06 (*p* = 0.303). The combined result remained outside the genome-wide threshold and was treated as descriptive only.

### 3.6. The F2 Locus Architecture Was Concordant in the FinnGen Subtraction Construct

The FinnGen subtraction analysis produced 1,259,947 factor estimates. FinnGen F2 had SNP heritability 0.00805 (s.e. 0.00139; Z = 5.81) and an LDSC intercept of 1.0037. Thirteen F2 lead SNPs were available as exact matches; rs12524544 and its eligible FUMA proxy set were unavailable.

**Figure 3:**
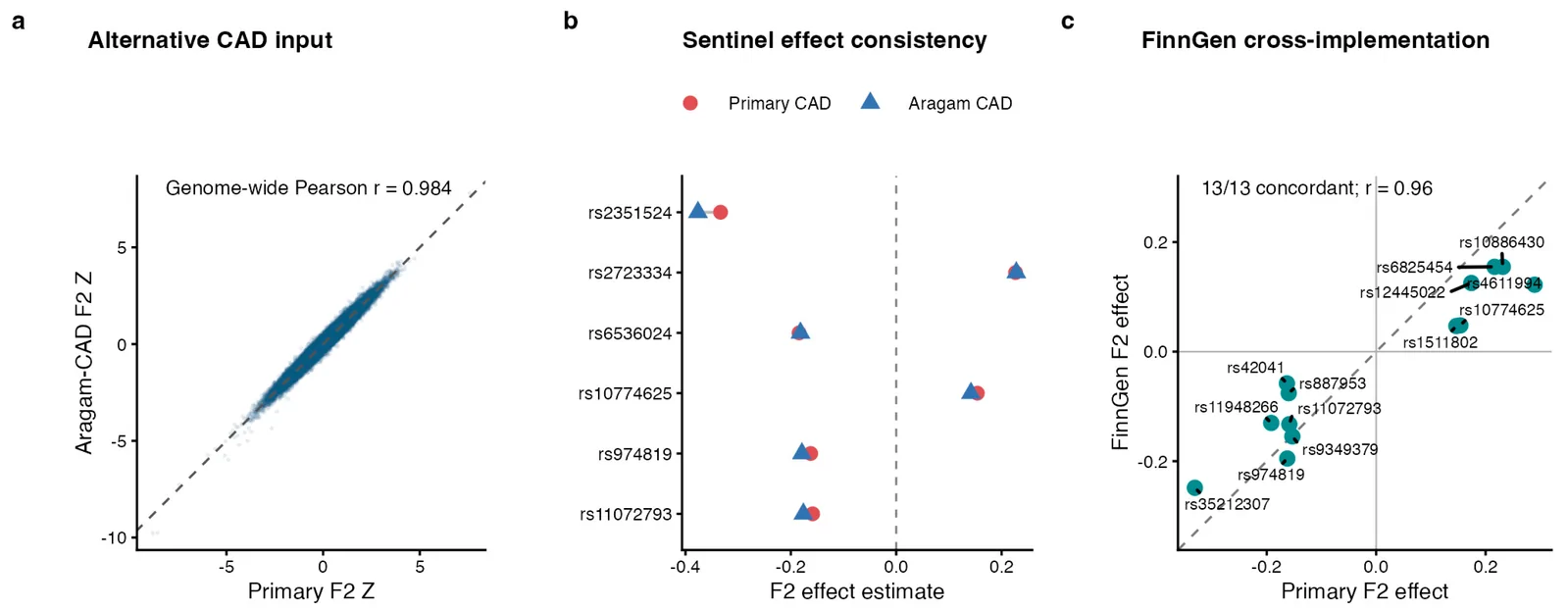
Robustness of the F2 architecture. (**a**) Genome-wide F2 Z scores from the primary and alternative-CAD analyses; the plot shows a deterministic sample of 80,000 variants and reports the correlation calculated across all matched valid variants. (**b**) Effect estimates for six prespecified sentinel variants after replacing the primary CAD GWAS with the Aragam CAD GWAS. (**c**) The primary analysis and FinnGen F2 effect estimates for 13 exact-matched lead variants. FinnGen is interpreted as a cross-implementation comparison with potential participant overlap because FinnGen contributed to GIGASTROKE.

All 13 tested lead SNPs had the same effect direction in the primary analysis and FinnGen. Direction consistency exceeded chance expectation (exact binomial *p* = 2.44 × 10^−4^). Ten variants had the same direction with *p* < 0.05, and nine met the *p* < 0.001 criterion (Table 6). rs974819 showed a concordant FinnGen F2 effect of −0.195 (s.e. 0.0317; *p* = 7.23 × 10^−10^). Given FinnGen’s contribution to the European GIGASTROKE meta-analysis, these results describe cross-implementation concordance in potentially overlapping samples (Figure 3).

### 3.7. F1 and F2 Showed Distinct Genetic Correlation Profiles

**Figure 4:**
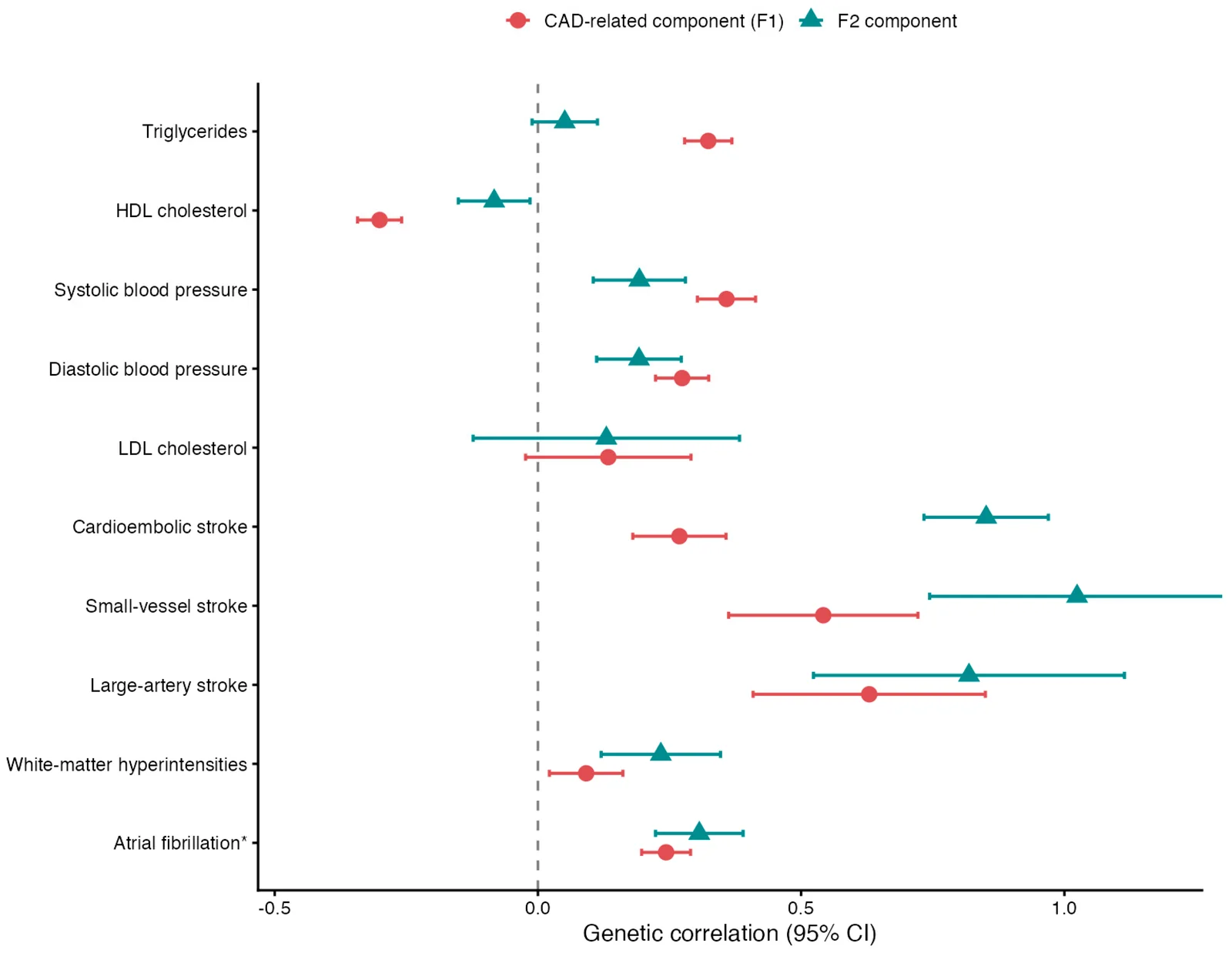
Genetic correlations of F1 and F2. Points show LDSC genetic correlations and bars show 95% confidence intervals. Red circles denote F1; teal triangles denote F2. Formal F1–F2 contrasts used the complete three-trait LDSC sampling covariance matrix and were corrected across nine prespecified traits. Atrial fibrillation, marked by an asterisk, was a secondary joint analysis with an unadjusted formal contrast. Candidate VTE/DVT analyses were excluded because the archived external files did not pass phenotype and sample-size provenance quality control.

Formal component contrasts passed nine-trait FDR correction for triglycerides, CES, HDL cholesterol, SBP and SVS (Figure 4 and Table 7). F1 correlated more strongly with triglycerides than F2 (0.324 versus 0.051; Delta = −0.273; FDR = 7.95 × 10^−12^). F1 also had a stronger positive correlation with SBP and a stronger negative correlation with HDL cholesterol.

Within the overlapping GIGASTROKE subtype analyses, F2 showed a stronger genetic correlation with CES than F1 (0.851 versus 0.269; Delta = 0.583; FDR = 1.45 × 10^−11^). The SVS contrast also passed FDR correction, but the F2 estimate exceeded one. SVS SNP heritability had Z = 3.11, so this boundary estimate remained exploratory. These CES, LAS and SVS estimates describe part-whole subtype alignment and were not treated as independent validation.

The F1–F2 differences were not significant after FDR correction for white matter hyperintensities, diastolic blood pressure, LAS or LDL cholesterol. These results distinguished a stronger triglyceride, HDL and SBP profile for F1 from a stronger CES profile for F2.

Both factors also correlated positively with AF in the joint secondary analysis: rg(F1,AF) = 0.243 (s.e. = 0.0237; *p* = 9.17 × 10^−25^) and rg(F2,AF) = 0.307 (s.e. = 0.0425; *p* = 5.34 × 10^−13^). The numerical difference was not significant in the formal covariance-aware contrast (Delta = 0.0631, s.e. = 0.0526; *p* = 0.230; Table S6). AF-related genetic liability therefore spanned both components, and the analysis did not resolve a between-component difference.

### 3.8. Functional Analyses Linked F1 to Lipid-Arterial Biology and F2 to Coagulation-Related Loci

MAGMA identified 242 Bonferroni-significant genes for F1. Seven GTEx tissues passed Bonferroni correction: coronary artery, aorta, tibial artery, uterus, fallopian tube, lung and endocervix. F1 also had 32 significant gene sets, led by LDL, HDL, triglyceride, cholesterol and lipoprotein pathways.

F2 contained 13 Bonferroni-significant MAGMA genes: WDR12, FGG, PMF1-BGLAP, PMF1, SLC25A44, ICA1L, FGA, ZNF474, CARF, KLKB1, F11, CYP4V2 and NFE2. No F2 GTEx tissue passed Bonferroni correction across 54 tissues. No F2 gene set passed Bonferroni correction across 17,001 tests.

In the paired local MAGMA v1.08 sensitivity analysis, changing only the constant SNP N from 1,296,908 to 236,506 left all 16,814 tested gene Z scores and *p* values unchanged (both correlations = 1.000). All 12 genes significant in this paired local workflow were retained, and FGA, FGG, F11 and KLKB1 retained their ranks and significance status (Table S15). The frozen online FUMA workflow tested 17,512 Ensembl-annotated genes and identified 13 significant genes; the local reproduction tested 16,814 NCBI37.3-annotated genes after SNP mapping and identified 12. Eleven significant gene symbols overlapped. NFE2 and ZNF474 were significant only in the online Ensembl-based output, whereas LOC100505841 appeared only in the local NCBI37.3 set. These differences reflect annotation identifiers and boundaries rather than the N sensitivity, which changed no result within the paired local workflow.

In targeted S-LDSC, tibial artery was the only tissue passing FDR correction, and only for F1 (*p* = 7.66 × 10^−4^; FDR = 0.0161). Coronary artery and aorta were nominally associated with F1. Tibial artery was nominally associated with F2 (*p* = 0.0277; FDR = 0.582). No F2 tissue passed FDR correction, and all 13 brain tissues were non-significant for both factors (Table S3).

### 3.9. Strict SMR and HEIDI Analyses Prioritised Two Arterial Expression Signals for F2

**Figure 5:**
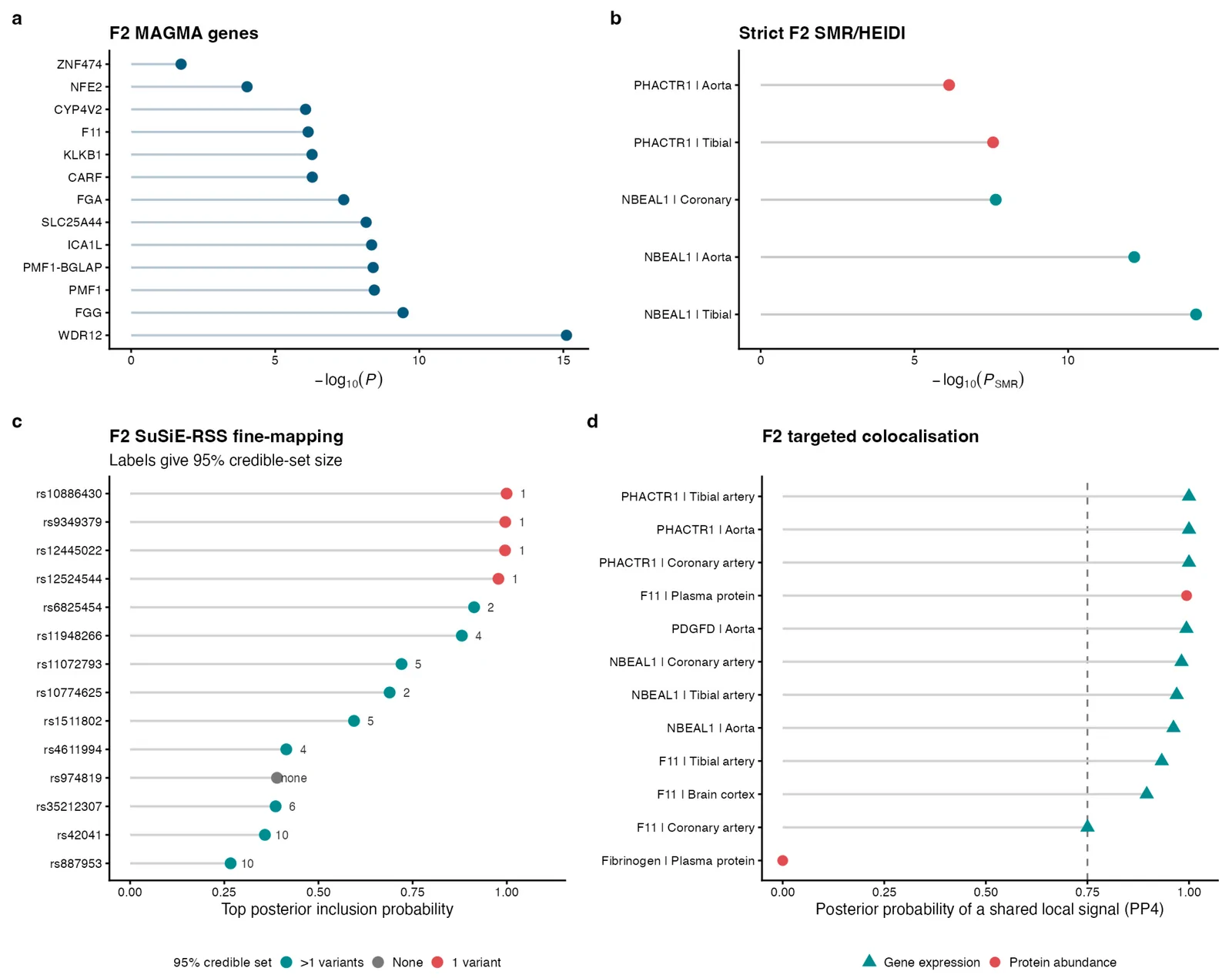
Molecular and regulatory characterisation of the F2 component. (**a**) Bonferroni-significant F2 MAGMA genes. (**b**) F2 arterial gene–tissue associations passing *p*(SMR) < 8.149 × 10^−7^ and *p*(HEIDI) > 0.01. (**c**) Maximum variant PIP at each of the 14 FUMA-defined F2 loci; labels give purity-qualified 95% credible-set size. (**d**) PP4 for selected eQTL and pQTL colocalisation results; the dashed line denotes PP4 = 0.75. These analyses prioritise compatible local signals for subsequent mechanistic testing.

Applying the global SMR threshold and HEIDI filter retained 73 F1 tissue–gene associations involving 38 unique genes. F2 retained five tissue–gene associations involving NBEAL1 and PHACTR1, all in arterial tissues (Figure 5 and Table 8). No brain expression association passed the strict F2 criteria.

NBEAL1 was associated with F2 in tibial artery (b(SMR) = 0.505; s.e. 0.0649; *p* = 6.87 × 10^−15^), aorta (b(SMR) = 0.428; *p* = 7.05 × 10^−13^) and coronary artery (b(SMR) = 0.560; *p* = 2.26 × 10^−8^). PHACTR1 passed the strict criteria in tibial artery (b(SMR) = 0.455; *p* = 2.76 × 10^−8^) and aorta (b(SMR) = 0.547; *p* = 7.43 × 10^−7^).

### 3.10. Colocalisation Supported Shared Local Regulatory Signals at Selected F2 Loci

Source-trait analyses placed the F2 colocalisation results in their input-GWAS context (Figure S1 and Table S13). The NBEAL1 aortic signal was high for AIS, CAD, F1 and F2 (PP4 = 0.976, 0.987, 0.987 and 0.962). PHACTR1 tibial-artery expression showed PP4 = 1.000 for CAD and F1 and 0.99999 for F2 but 0.005 for AIS. PDGFD aortic expression similarly colocalised with CAD, F1 and F2 (PP4 = 0.994, 0.994 and 0.994) more strongly than with AIS (PP4 = 0.307). These patterns indicate that high F2 PP4 at NBEAL1, PHACTR1 and PDGFD was not unique to the residual construct.

At the protein level, F11 abundance showed high PP4 with AIS (0.999) and F2 (0.994) but not with CAD (0.00137) or F1 (0.00295). F11 tibial-artery expression was also strongest for F2 (PP4 = 0.933), whereas the corresponding AIS result was intermediate (0.505). The FGA/FGG region showed distinct F2 and fibrinogen protein signals (PP3 > 0.99999998; PP4 = 3.78 × 10^−9^) (Table 9). The complete 38-combination source-context analysis is reported in Table S13.

## 4. Discussion

GWAS-by-subtraction separated the AIS association profile into two statistically defined components. F1 captured effects shared with CAD and recovered the broad polygenic and cardiometabolic profile of coronary disease. F2 retained heritable AIS variation after that shared architecture was modelled. Locus definition, covariance-aware genetic-correlation contrasts, functional annotation and FinnGen reimplementation showed that the distinction persisted across analytic perspectives. The analysis extends the multivariate use of Genomic SEM [9] and GWAS-by-subtraction [10] to two clinically overlapping arterial outcomes.

The sensitivity analyses quantify both stability and model dependence. F2 Z scores remained highly correlated across negative and positive factor covariances, population-prevalence settings and the alternative CAD input. Reverse ordering produced the expected reassignment of shared and residual signal: forward F2 aligned strongly with the AIS-first shared construct, while the reverse residual-CAD construct showed an inverse relationship. The result supports F2 as a stable residual contrast within the prespecified CAD-first model, not an order-invariant stroke subtype. LD-reference sensitivity preserved the top variant at four of five key loci and retained PHACTR1 as a single-variant credible set, while F11/KLKB1 remained reference-sensitive.

F1 recovered the expected coronary genetic architecture. Its larger heritability, 138 FUMA loci and 242 significant MAGMA genes reflect the extensive polygenicity reported by successive CAD GWAS [5,6]. Stronger correlations with triglycerides and systolic blood pressure, together with a more negative HDL-cholesterol correlation, localised F1 to cardiometabolic architecture shared by AIS and CAD. This profile agrees with evidence that lipid effects are strongest for atherosclerotic stroke mechanisms [7] and that blood pressure contributes across ischaemic stroke definitions [8]. Arterial signals also aligned across MAGMA, targeted S-LDSC and SMR, complementing CAD studies that identified arterial-wall regulatory mechanisms [30]. The agreement across analyses shows that F1 retained established CAD-related structure.

F2 showed a different profile. Its genetic correlation with cardioembolic stroke was substantially stronger than that of F1, whereas the triglyceride, HDL-cholesterol and systolic-blood-pressure contrasts favoured F1. The 13 significant F2 MAGMA genes included the fibrinogen genes FGA and FGG and the intrinsic-pathway genes F11 and KLKB1. Fine-mapping separated regional resolution from gene-level interpretation: the FGA/FGG region had a two-variant credible set led by rs6825454, whereas the F11/KLKB1 region retained five correlated candidates led by rs2289252. F11 protein abundance shared a local association pattern with F2, while circulating fibrinogen showed strong support for a distinct regional signal. Earlier candidate-gene work linked FGA/FGG haplotypes to ischaemic stroke independently of measured fibrinogen concentration [31]. More recent Mendelian-randomisation studies associated genetically proxied reductions in fibrinogen chains and factor XI with lower AIS or cardioembolic-stroke risk [32,33]. The stronger CES correlation and regional evidence converge on a coagulation-related branch of F2 while keeping gene-level and circulating-protein evidence distinct.

The stronger F2 correlation with cardioembolic stroke also fits a component less dominated by coronary atherosclerosis. Large AF GWAS demonstrate a highly polygenic arrhythmia architecture [23,34], and AF polygenic risk can discriminate cardioembolic from non-cardioembolic stroke beyond clinical predictors [35]. Our secondary analysis found positive AF correlations for both factors. Although the F2 estimate was numerically higher, the formal F2-F1 contrast was not significant. AF-related liability thus appears across both components. F2 also contained established stroke regions near FOXF2, PHACTR1, GRK5, SH2B3 and NBEAL1; dedicated lacunar and cerebral small-vessel disease GWAS place several of these signals within broader cerebrovascular architecture [4,36].

The 11q22.3 association illustrates the type of signal exposed by subtraction. rs974819 reached genome-wide significance for F2 despite a conventional GIGASTROKE AIS *p* value of 5.17 × 10^−4^. The region was previously associated with CAD [37,38], and the original C4D study reported rs974819 as a PDGFD eQTL in aortic media, with concordant suggestive associations in aortic adventitia and mammary artery [39]. In our aligned data, the T allele increased CAD risk but had negative AIS effects in GIGASTROKE and FinnGen. This directional discordance matches the contrast targeted by the model and provides a statistical explanation for emergence of the signal after subtraction. Fine-mapping assigned the largest PIP to rs974819 but produced no purity-qualified 95% credible set. By contrast, aortic PDGFD expression showed a strong shared local association pattern with F2 (PP4 = 0.994), prioritising a regulatory hypothesis without resolving the regional variant. Archived and current Catalog queries and direct regional scans found no prior genome-wide significant stroke association for the lead, its high-LD proxies, or the surrounding region. We therefore classify this finding as a previously unreported genome-wide significant F2 association at an established CAD locus, not as a new vascular region or a directly replicated AIS locus.

The aligned source-trait table clarifies this result. The T allele had positive CAD estimates and negative marginal AIS estimates in both implementations, so the significant F2 coefficient is a model-derived CAD-AIS discordant association. Its sign does not establish a protective effect on AIS.

Reimplementation in FinnGen showed concordance of the F2 architecture in a later release and under a different endpoint definition [11]. All 13 available lead variants had concordant directions, including rs974819, and most passed the prespecified graded *p*-value criteria. FinnGen also contributed 8851 cases and 174,426 controls to the European GIGASTROKE meta-analysis [3], so cohort overlap could explain part of the agreement. These data support cross-implementation stability of the subtraction component within potentially overlapping samples.

Tissue-enrichment analyses did not isolate a specific F2 tissue. No F2 tissue passed Bonferroni correction in MAGMA or FDR correction in targeted S-LDSC, and all tested brain annotations were non-significant. These methods localise relative heritability enrichment and have limited sensitivity to distributed or cell-state-specific effects [24]. F2 is therefore best viewed as a distributed architecture spanning vascular, haemostatic, cardiac and cerebral–vascular processes.

Expression-informed analysis prioritised NBEAL1 and PHACTR1 for F2 in arterial tissues. NBEAL1 lies within a region implicated by stroke and cerebral small-vessel disease GWAS [3,36]. PHACTR1 has context-dependent associations across coronary, migraine, cervical-artery dissection and fibromuscular dysplasia phenotypes, with rs9349379 experimentally linked to distal regulation of EDN1 [40]. Both genes passed the global SMR threshold in more than one artery and retained high arterial PP4 values in targeted colocalisation. The PHACTR1 result aligned with its single-variant SuSiE credible set, whereas NBEAL1 remained a multivariant region despite consistent arterial expression evidence. This agreement places NBEAL1 and PHACTR1 among the leading regulatory candidates in our F2 analysis.

Source-trait colocalisation further separated inherited input signals from residual-construct patterns. NBEAL1 was shared across both source traits and both factors. PHACTR1 and PDGFD were prominent in CAD, F1 and F2, whereas the F11 protein signal aligned with AIS and F2. The molecular results therefore define source-signal patterns rather than a set of associations unique to F2.

The decomposition was evaluated at several levels. The primary model incorporated the complete multivariable LDSC sampling covariance matrix [14], all factor GWAS models converged and covariance sensitivity analysis showed high genome-wide stability. Replacing the CAD input preserved all six prespecified sentinel signals. Locus counts came from frozen online FUMA jobs [17], while novelty assessment combined Catalog queries with direct scans of regional stroke summary statistics. Fine-mapping used allele-aligned signed LD, and genetic-correlation contrasts, including the secondary AF test, used joint three-trait LDSC matrices with the verified sampling-covariance order. These checks reduce dependence on any single input or inferential step.

Several limitations define the scope of the model. The analysis used European-ancestry summary statistics, and F2 is conditional on the CAD-first Cholesky order. The reverse-order analysis quantified this dependence, while covariance, prevalence and alternative-CAD analyses assessed stability within that definition. FinnGen participation in GIGASTROKE limits the comparison to cross-implementation concordance. FUMA mapping was positional. SuSiE-RSS was restricted to the HapMap3 variants carried by the factor GWAS; even the larger UK Biobank LD reference could not evaluate absent variants. No conditional association analysis was performed, so loci may contain multiple signals. The conservative N used for MAGMA was a sensitivity parameter rather than a factor effective-sample-size estimate. Targeted colocalisation assumed at most one causal variant per trait per region and compared statistically dependent source and factor GWAS descriptively. Expression-informed analyses prioritise candidate transcripts and shared local signals rather than establish transcript-level causality.

## 5. Conclusions

GWAS-by-subtraction separated a highly polygenic CAD-related component from a smaller heritable, model-defined residual AIS component. F2 remained stable across covariance, prevalence and CAD-input sensitivities, while reverse ordering quantified its dependence on the CAD-first specification. The aligned rs974819 effects exposed a CAD-AIS discordant association rather than a protective AIS effect. Source-trait colocalisation showed that several regulatory signals were inherited from one or both inputs, whereas the F11 protein signal aligned with AIS and F2. Together, these results support a component-based view of AIS genetic effects after modelling shared coronary architecture.

## Figures and Tables

**Table 2:** Factor GWAS and FUMA summary.

Comp	h2 (s.e.)	Z	Int.	GW SNPs	Indep. SNPs	Lead SNPs	Risk Loci	Cand. SNPs	Pos. Genes	MAGMA Genes	GTEx n
F1	0.02509 (0.00175)	14.33	0.9916	2278	503	215	138	20,980	449	242	7
F2	0.004144 (0.000418)	9.91	1.0047	96	24	14	14	1085	42	13	0

**Table 3:** Fourteen FUMA-defined lead loci for F2.

Locus	Lead SNP	Position	A1/A2	EAF	Beta	s.e.	*p*(F2)	*p*(AIS)	Mapped Genes	Novelty
1	rs887953	1:156,191,149	C/T	0.3417	−0.1598	0.0259	6.69 × 10^−10^	3.43 × 10^−11^	BGLAP; PAQR6; PMF1; PMF1-BGLAP; SEMA4A; SLC25A44	Known stroke locus
2	rs35212307	2:203,765,756	C/T	0.1244	−0.3312	0.0376	1.24 × 10^−18^	9.80 × 10^−10^	ABI2; CARF; CYP20A1; FAM117B; ICA1L; NBEAL1; WDR12	Known stroke locus
3	rs4611994	4:111,711,041	C/T	0.1269	0.2895	0.0368	3.89 × 10^−15^	1.87 × 10^−19^	None	Known stroke locus
4	rs6825454	4:155,501,188	C/T	0.2723	0.2163	0.0276	5.05 × 10^−15^	1.47 × 10^−16^	FGA; FGB; FGG; LRAT; PLRG1	Known stroke locus
5	rs1511802	4:187,150,806	C/T	0.3974	0.1466	0.0252	5.83 × 10^−9^	7.73 × 10^−8^	AC110771.1; CYP4V2; F11; KLKB1	Known stroke locus
6	rs11948266	5:121,522,316	C/T	0.1685	−0.1918	0.0339	1.61 × 10^−8^	1.95 × 10^−9^	ZNF474	Known stroke locus
7	rs12524544	6:1,366,220	C/T	0.1165	0.2969	0.0405	2.23 × 10^−13^	1.14 × 10^−13^	FOXF2; FOXQ1	Known stroke locus
8	rs9349379	6:12,903,957	G/A	0.4152	−0.1531	0.0251	1.12 × 10^−9^	0.148	PHACTR1	Known stroke-related locus
9	rs42041	7:92,246,744	G/C	0.2470	−0.1631	0.0294	2.91 × 10^−8^	3.61 × 10^−11^	CDK6	Known stroke locus
10	rs10886430	10:121,010,256	G/A	0.1240	0.2314	0.0404	9.97 × 10^−9^	1.07 × 10^−11^	GRK5	Known stroke locus
11	rs974819	11:103,660,567	T/C	0.2704	−0.1624	0.0285	1.16 × 10^−8^	5.17 × 10^−4^	None	No prior stroke signal; known CAD locus
12	rs10774625	12:111,910,219	A/G	0.4789	0.1538	0.0251	8.25 × 10^−10^	1.22 × 10^−19^	ACAD10; ALDH2; ATXN2; BRAP; HECTD4; MAPKAPK5; NAA25; PTPN11; RPL6; SH2B3; TRAFD1	Known stroke locus
13	rs11072793	15:79,006,442	G/A	0.2832	−0.1587	0.0282	1.74 × 10^−8^	4.97 × 10^−4^	ADAMTS7; CHRNB4	Known stroke locus
14	rs12445022	16:87,575,332	A/G	0.3292	0.1739	0.0272	1.74 × 10^−10^	9.14 × 10^−11^	None	Known stroke locus

**Table 4:** Fine-mapping summary for three mechanism-relevant F2 loci.

Region	Lead SNP	95% Credible Set	Top SNP	PIP
11q22.3 CAD-discordant locus	rs974819	None purity-qualified	rs974819	0.390
FGA/FGG fibrinogen locus	rs6825454	2 variants	rs6825454	0.913
F11/KLKB1 locus	rs1511802	5 variants	rs2289252	0.594

**Table 5:** Allele-aligned rs974819 effects across source traits and subtraction constructs.

Dataset or Construct	Cohort	Beta (T)	s.e.	*p*	T-Allele Frequency	Effect Scale
Primary CAD	UKB/CARDIoGRAMplusC4D	0.0614	0.0055	1.12 × 10^−28^	0.3082	Log-odds
Primary AIS	GIGASTROKE European	−0.0282	0.0081	5.17 × 10^−4^	0.2704	Log-odds
Primary F1	Primary model	0.1212	0.0109	6.21 × 10^−29^	0.2704	Latent coefficient
Primary F2	Primary model	−0.1624	0.0285	1.16 × 10^−8^	0.2704	Latent coefficient
Aragam CAD	Alternative CAD	0.0623	0.00538	5.42 × 10^−31^	NA	Log-odds
Alternative-CAD F1	Alternative-CAD model	0.1420	0.0123	7.21 × 10^−31^	0.2704	Latent coefficient
Alternative-CAD F2	Alternative-CAD model	−0.1789	0.0289	5.63 × 10^−10^	0.2704	Latent coefficient
FinnGen stroke	FinnGen R13	−0.0414	0.00988	2.84 × 10^−5^	0.2145	Log-odds
FinnGen CHD	FinnGen R13	0.0505	0.00701	5.88 × 10^−13^	0.2144	Log-odds
FinnGen F2	FinnGen model	−0.1951	0.0317	7.23 × 10^−10^	0.2145	Latent coefficient

**Table 6:** FinnGen cross-implementation comparison of F2 lead variants.

Lead SNP	Primary Beta	Primary *p*	FinnGen Beta	FinnGen *p*	Same Sign	Same Sign; *p* Below 0.001
rs887953	−0.1598	6.69 × 10^−10^	−0.0758	5.98 × 10^−3^	Yes	No
rs35212307	−0.3312	1.24 × 10^−18^	−0.2486	1.51 × 10^−9^	Yes	Yes
rs4611994	0.2895	3.89 × 10^−15^	0.1221	7.97 × 10^−4^	Yes	Yes
rs6825454	0.2163	5.05 × 10^−15^	0.1548	2.18 × 10^−8^	Yes	Yes
rs1511802	0.1466	5.83 × 10^−9^	0.0468	0.0725	Yes	No
rs11948266	−0.1918	1.61 × 10^−8^	−0.1302	1.02 × 10^−4^	Yes	Yes
rs12524544	0.2969	2.23 × 10^−13^	NA	NA	NA	NA
rs9349379	−0.1531	1.12 × 10^−9^	−0.1548	2.27 × 10^−9^	Yes	Yes
rs42041	−0.1631	2.91 × 10^−8^	−0.0580	0.0578	Yes	No
rs10886430	0.2314	9.97 × 10^−9^	0.1548	5.68 × 10^−4^	Yes	Yes
rs974819	−0.1624	1.16 × 10^−8^	−0.1951	7.23 × 10^−10^	Yes	Yes
rs10774625	0.1538	8.25 × 10^−10^	0.0480	0.0656	Yes	No
rs11072793	−0.1587	1.74 × 10^−8^	−0.1326	7.67 × 10^−7^	Yes	Yes
rs12445022	0.1739	1.74 × 10^−10^	0.1253	6.31 × 10^−6^	Yes	Yes

**Table 7:** Formal contrasts of genetic correlations.

Trait	rg(F1), s.e.	rg(F2), s.e.	F2 − F1	*p*	FDR	Summary
Triglycerides	0.324 (0.0229)	0.051 (0.0317)	−0.273	8.83 × 10^−13^	7.95× 10^−12^	F1 stronger
Cardioembolic stroke	0.269 (0.0452)	0.851 (0.0603)	0.583	3.23 × 10^−12^	1.45 × 10^−11^	F2 stronger
HDL cholesterol	−0.301 (0.0214)	−0.083 (0.0348)	0.218	2.16 × 10^−7^	6.49 × 10^−7^	F1 more negative
Small-vessel stroke	0.542 (0.0917)	1.024 (0.1429)	0.482	2.63 × 10^−4^	5.92 × 10^−4^	Exploratory boundary estimate
Systolic blood pressure	0.358 (0.0282)	0.193 (0.0447)	−0.165	0.00228	0.00411	F1 stronger
White matter hyperintensities	0.092 (0.0356)	0.234 (0.0579)	0.142	0.0528	0.0792	Difference not significant
Diastolic blood pressure	0.274 (0.0258)	0.192 (0.0410)	−0.0819	0.0886	0.114	Difference not significant
Large-artery stroke	0.629 (0.1126)	0.819 (0.1507)	0.190	0.220	0.247	Difference not significant; low h2 Z
LDL cholesterol	0.134 (0.0802)	0.130 (0.1290)	−0.00368	0.981	0.981	Difference not significant

Delta was defined as rg(F2) − rg(F1).

**Table 8:** Strict F2 SMR and HEIDI associations.

Gene	Tissue	Top SNP	b(SMR)	s.e.	*p*(SMR)	*p*(HEIDI)	HEIDI SNPs
NBEAL1	Artery tibial	rs2351524	0.505	0.0649	6.87 × 10^−15^	0.705	20
NBEAL1	Artery aorta	rs4675310	0.428	0.0596	7.05 × 10^−13^	0.821	20
NBEAL1	Artery coronary	rs4675310	0.560	0.100	2.26 × 10^−8^	0.0231	5
PHACTR1	Artery tibial	rs9349379	0.455	0.0819	2.76 × 10^−8^	0.575	20
PHACTR1	Artery aorta	rs9349379	0.547	0.110	7.43 × 10^−7^	0.366	19

**Table 9:** Selected F2 colocalisation results.

Locus or Target	QTL Phenotype	Tissue or Source	SNPs	PP3	PP4
NBEAL1	Gene expression	Aorta/coronary/tibial artery	265–266	0.018–0.038	0.962–0.982
PHACTR1	Gene expression	Aorta/coronary/tibial artery	556	8.33 × 10^−6^–7.48 × 10^−5^	0.99990–0.99999
PDGFD	Gene expression	Aorta	572	0.00624	0.994
F11	Gene expression	Tibial artery	602	0.0670	0.933
F11	Gene expression	Brain cortex	602	0.0788	0.896
F11	Protein abundance	GCST90469165	604	0.00595	0.994
Fibrinogen	Protein abundance	GCST90019421	420	>0.99999998	3.78 × 10^−9^

## Data Availability

The data analysed in this study were derived from public resources. AIS and CAD summary statistics are available from the NHGRI-EBI GWAS Catalog under GCST90104540 and GCST005194. FinnGen Release 13 summary statistics for stroke excluding subarachnoid haemorrhage and major coronary heart disease events are available through the FinnGen Results portal. GTEx v8 cis-eQTL data are available from the GTEx Portal. F11 protein and fibrinogen summary statistics are available under GCST90469165 and GCST90019421. The revision package includes analysis code, build instructions, manifests and SHA-256 checksums. The public repository DOI will be inserted after author upload: [REPOSITORY DOI PENDING]. Source-GWAS redistribution terms apply to raw input files.

## Supplementary Materials

The following supporting information can be downloaded at: https://www.mdpi.com/article/10.3390/genes17091061/s1, Table S1, extended F1–F2 covariance sensitivity; Table S2, GWAS sources for external traits and overlapping stroke-subtype analyses; Table S3, S-LDSC results; Table S4, strict F1 SMR and HEIDI associations; Table S5, effective sample sizes; Table S6, atrial-fibrillation genetic correlations; Table S7, alternative-CAD sensitivity; Table S8, signed-LD SuSiE-RSS fine-mapping; Table S9, targeted F2-QTL colocalisation; Table S10, reverse-Cholesky order sensitivity; Table S11, population-prevalence sensitivity; Table S12, complete rs974819 aligned effects; Table S13, source-trait colocalisation; Table S14, LD-reference sensitivity; Table S15, MAGMA sample-size sensitivity; Figure S1, source-trait context for selected colocalisation results.

## Abbreviations

The following abbreviations are used in this manuscript:

AF	atrial fibrillation
AIS	any ischaemic stroke
API	application programming interface
CAD	coronary artery disease
CES	cardioembolic stroke
CHD	coronary heart disease
DVT	deep-vein thrombosis
EAF	effect-allele frequency
eQTL	expression quantitative trait locus
FDR	false discovery rate
FUMA	Functional Mapping and Annotation
GWAS	genome-wide association study
HDL	high-density lipoprotein
HEIDI	heterogeneity in dependent instruments
INFO	imputation quality score
LAS	large-artery stroke
LD	linkage disequilibrium
LDL	low-density lipoprotein
LDSC	LD score regression
MAGMA	Multimarker Analysis of GenoMic Annotation
MHC	major histocompatibility complex
Neff	effective sample size
PIP	posterior inclusion probability
pQTL	protein quantitative trait locus
PP3	posterior probability of distinct local signals
PP4	posterior probability of a shared local signal
QTL	quantitative trait locus
S-LDSC	stratified LD score regression
SBP	systolic blood pressure
SEM	structural equation modelling
SMR	summary-data-based Mendelian randomisation
SNP	single-nucleotide polymorphism
SVS	small-vessel stroke
SuSiE-RSS	sum of single effects regression using summary statistics
UK	United Kingdom
VTE	venous thromboembolism
